# The great dispersal: The fall and rise of global environmental governance

**DOI:** 10.1007/s13280-025-02177-x

**Published:** 2025-05-03

**Authors:** Sverker Sörlin, Paul Warde, Isobel Akerman, Jasmin Höglund Hellgren, Sabine Höhler, Erik Isberg, Eric Paglia, Gloria Samosír, Thomas Harbøll Schrøder

**Affiliations:** 1 Division of History of Science, Technology and Environment, KTH Royal Institute of Technology, Teknikringen 74D, 100 44 Stockholm, Sweden; 2 Center for History and Economics, and Faculty of History, University of Cambridge, West Road, Cambridge, CB3 9EF UK; 3 Faculty of History, University of Cambridge, West Road, Cambridge, CB3 9EF UK

**Keywords:** Diplomatic history, Earth system science, History, Environmental governance, History, Environmental history, Global environmental governance, Governable environmental objects

## Abstract

This article presents a new way of understanding Global Environmental Governance (GEG), historically and functionally. We outline a revised analytical framing, which connects the post-WWII moment of early globalizing conservation with the intensifying attempts to govern the human-earth relationship through an ever-growing assemblage of *governable environmental objects* and their quantifiable indicators as *proxies*. Our argument is as follows: (1) GEG has followed a *trajectory of dispersal* of actors, institutions, conceptual tools and responsibilities from the micro- and local scales to the planetary. We analyze how these trajectories unfold in three essential domains: Earth System science, sovereignty, and neoliberalization. (2) GEG *is performative*. The governance itself has created the dynamic environmental objects under governance. (3) In this way, GEG has *normalized the environment* as a policy object.

## Introduction—reframing global environmental governance

There is hardly reason for optimism. The world is divided into sovereign states that are not willing to give up their formal freedom of action. It is one of the ironies of history that the principle of national sovereignty and equality received its triumphal confirmation in the Charter of the United Nations at the time when the introduction of atomic weapons, the development of communications, the rapid industrialization and the awakening consciousness of the environmental risks made it unmistakably clear that all of humanity is interdependent and that the old concept of sovereignty is inadequate (Åström 1972).

**Fig. 1:**
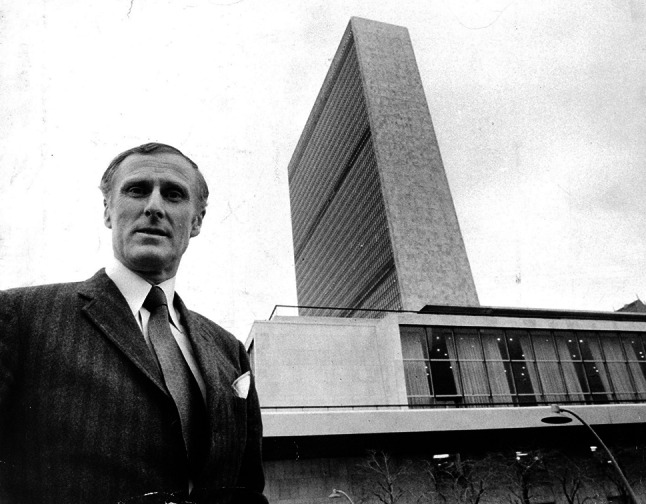
Top diplomat Sverker Åström, Sweden’s ambassador to the United Nations 1964 to 1970

So lamented Ambassador Sverker Åström, one of the main architects of the UN Conference on the Human Environment, in the first issue of the journal *Ambio* published four months before the June 1972 event that brought representatives of 114 countries to Stockholm for the world’s first comprehensive environmental conference on a global scale. This might seem a concise diagnosis of what the world needed. To meet environmental challenges, the world must develop a new form of global governance that sets aside national sovereignty and self-interest. A Global Environmental Governance. This, however, is not what happened. One of the main points of agreement at the Stockholm conference was the assertion of absolute national sovereignty over resources (and resources were the category through which the environment was largely understood). A global environmental government fell before it even had the chance to walk (Fig. 1).

And yet, fifty years later, we find… Global Environmental Governance, GEG for short. It has proven relatively easy to define on a conceptual level, but much harder to operationalize (Biermann and Pattberg 2008). It is not even obvious who engages or participates in it. An umbrella concept for the management of the global environment, GEG encompasses a wide range of practices and ideas, including negotiations such as the Conference of Parties meetings (COP), and agreements such as the 1997 Kyoto Protocol on reducing greenhouse gas emissions. The GEG concept also captures regulations, their underlying ideas, data and scientific principles such as the precautionary principle and the polluter pays principle in environmental law, as well as their institutions and organizations, be they legal, diplomatic, political or other. Global environmental governance, by most definitions, is a supra-national activity encompassing multiple state and other actors (UNEP 2023). This is historically significant, and notable achievements have been made through the apparatus that GEG comprises (Ivanova 2021).

Yet if compared to the magnitude of the enterprise, especially the degree of governance and coordination needed to reach sustainability goals, what has been achieved so far seems less impressive, and might even be characterized as a failure, which is an almost ritualistic feature in the literature on global environmental politics (e.g., Kramerz and Parker 2016). The UN Sustainable Development Goals (SDG) from 2015, including the 1.5 °C climate goal of the Paris agreement, have made marginal progress and are unlikely to be met during the Agenda 2030 decade (UN 2023). The underwhelming results of the two-week COP 28 conference of the UNFCCC in Dubai in December 2023, attended by some 84,000 officials, activists and lobbyists, can be seen as a testament to this overall lack of real progress despite a great deal of international attention on climate, environment and sustainable development issues.

This, however, is not the kind of judgment we want to make. Rather, we wish to take a radically different view of global environmental governance and emphasize its character as an evolving historical phenomenon. There is no doubt that something we call by this name, however insufficient, has existed for a long time and that it will continue to evolve, even if under mounting geopolitical stress. Instead of taking a binary approach on investigating whether there is a functioning GEG or not, we want to pose a more fundamental question: What is GEG, how has it emerged and evolved, and how is it perpetuated? We will argue that a historical perspective provides an insight into why GEG looks the way it does today, and why the environment has become a theme on almost all levels of decision making both internationally, transnationally, nationally, locally as well as both in the public and private spheres. Such a perspective suggests that GEG is less intentional, but more dynamic than might be expected. Herein lies the core message of this synthesis article. We argue that one of the most characteristic developments of GEG is the *dispersal* of actors and responsibilities. This *proliferation* of stakeholders and institutions, and dissemination of parameters, categories and indicators, as well as scientific follow-up and economic trade-off mechanisms have resulted in the *normalization* of the environment as a cohesive policy object.

### The analytical structure of this article

What we offer in this article is a reframed version of the governance history that puts emphasis on factors that can explain changes in governance and ultimately identify processes of transformation. It is worth noticing that GEG was not conceived, let alone conceptualized when ‘the environment’ started its remarkable career as a modern political idea in the immediate postwar years (Selcer 2018; Warde et al. 2018). This fact alone, speaks to the strong historicity of the environment. A substantive expansion came only in the 1970s, reflected also in a rapid growth of ‘environmental’ journals across virtually all fields of knowledge, including *Ambio*, launched in Stockholm in 1972 as one of the world’s early environmental journals (Sörlin 2021) and soon regarded as a voice from one of the key global venues of the new and expanding policy arena (Sörlin and Paglia 2025).

Despite the many efforts to state the obvious, namely that Global Environmental Governance is both necessary and viable, it is articulated in a paradoxical way: something that clearly exists in some, or many forms, but that simultaneously is lacking and hard to describe. This is why we will here attempt a different approach in order to understand why that is. In the subsequent sections, we will, in a first step, present our readings of governance and environment as the two major historically co-evolving elements of GEG, before, in a second step, identifying three historical trajectories of science, politics and economics as crucial for the global development of environmental governance post-WWII: Earth System science, sovereignty, and neoliberalization.

A set of cornerstone concepts hold our narrative together. These are: *normalization*, *governable environmental objects*, *proxy*, and *post-nature*. Alongside with *dispersal*—that captures our overarching idea—they reflect central features in a historical arc. This arc connects the postwar early moment of globalizing conservation with the intensifying attempts in the last half-century to govern the human-earth relationship through an ever-growing assemblage of governable environmental objects, in many ways unintentional and with fascinating histories of their own. These objects were understood via quantifiable indicators as proxies (Mulvin 2021) such as a ceiling value of global temperature or other ‘planetary boundaries’ (Steffen et al. 2015a), of a vulnerable earth system. It is no coincidence that this history dovetails with what in the Anthropocene literature is called ‘the Great Acceleration’ (McNeill and Engelke 2016). Acknowledging the recent planetary dominance of human agency, we argue that a governance by proxy has become the norm as the global human enterprise is, again largely unintentionally sliding into a post-nature state of environment and climate (Purdy 2015). While normalized in a dispersed and far from sufficient way, this post-nature version of the human-earth relationship is still searching ways forward in an often hostile and anthropocentric geopolitical climate. In conclusion, we therefore outline some possible, often competing, narrative pathways of GEG in the future. Finally, we also discuss some possible implications of such a reformed view of environmental governance, including a mobilization of concerns for what we might call a nature made more social, and moral. Two hopefully inspiring consequences of GEG’s conditions and effects of dispersal.

Dispersal is a word for a wider, but not an even distribution. It is not a diffusionist idea, of a spreading sameness, as advocated for example in diffusionist theories of cultures. It is, quite to the contrary, the potential for many actors, on all levels from the local to the global and planetary, to participate and generate dissimilarity within a particular category of phenomena, such as environmental institutions or regulations, and to do so at varying speed and intensity. Human governance of the environment has typically been seen as the outcome of a relation between governing institutions and the governed object, ‘the environment’. This approach to governance is usually and very usefully analyzed by the political, legal and economic sciences, which reflect on ‘environmental’ issues in subfields of environmental assessment studies, environmental policy studies, environmental economics, or as the object of international relations.

What is missing here, we argue, is an understanding of how the environment has not just been the object of negotiations but also their *effect*. This is where new technologies of power have their significance: they govern, but they are also implicated in the emergence of new governable environmental objects. The concept of governable environmental objects has roots in Foucault’s *gouvernementalité*, but is now more commonly used to understand how nature has, by human agency, transformed not just into an object of physical terraforming but also an object of measuring and governing (Foucault 1991/1978; Aronowsky 2018; Sörlin and Wormbs 2018). The environment of today is not the same as the environment imagined half a century ago. It is a product of human ‘impacts’, but not simply that; the environment we imagine and grapple with through GEG is actually shaped and conceptualized through the process of environmental governance itself. Our point resembles a move within science and technology (STS) studies toward the study of ‘enactment’ rather than ‘representation’. Material-semiotic practices such as GEG do not simply represent the environment but *perform it* (Law and Mol 2008; Lien and Law 2011; Woolgar and Lezaun 2013; van Wichelen 2019). This necessitates, we argue, a very different understanding of a ‘real’ GEG, the global environmental governance that became the historical and present reality rather than the putatively dysfunctional ideal.

## GEG—steps toward the concept

Global Governance has been discussed in political science and its subfield of International Relations (IR) since the early to mid-1990s, when “the course of history … [was] at a turning point” (Rosenau and Czempiel 1992, p. 1). At the end of the Cold War, “globalization” became the shorthand for a new world order marked by global financial, trade, transport and communication systems, market deregulation and privatization. As a result of these structural changes, previous theoretical “simplifying assumptions” of unitary, instrumental nation states and of the distribution of power among them as the primary organizing factor no longer proved apt for accurate analysis and understanding of the emerging order in world politics (Avant et al. 2010, p. 4). In turn, Global Governance marked the associated political structures and actors, decision-making bodies like the UN, the World Bank, the WTO, and proliferating NGOs that characterized the new “global empire” (Hardt and Negri 2000).

Notably preceding the global agency of neoliberal markets, Global *Environmental* Governance (GEG) denoted seeking global solutions to environmental problems like acid rain, the ozone hole, biodiversity loss, and climate change since the 1970s (Jasanoff and Martello 2004; Miller and Edwards 2001). In its first definitions, global environmental governance was predicated on an existing and identifiable international *community* and its *shared* environmental problems (O’Neill 2009, p. 4). Global environmental governance was envisioned as an international agency led by nation states and informed by scientific experts. The driving force of environmental governance was international diplomacy, with the 1987 Montreal Protocol and the 1997 Kyoto Protocol as examples of internationally binding multilateral environmental agreements. The notion of a central and coordinated authority lay behind the formation in 1972 of The United Nations Environment Program UNEP, which some would have liked to be a more powerful institution and did not cease to bring disappointment in its first several decades (Ivanova 2010). The idea of a reinforced international institution returned around 2000 in the debate around a new, or at least reformed UN-based “World Environment Organization” with more resources and decision-making authority (e.g., Biermann 2000, 2001), but met considerable resistance (e.g., Najam 2003) and could not surmount the then strong centrifugal forces of dispersing authority.

Against the notion of global governance smoothly operating on common problems, as organizations like the UN and UNEP seemed to imply, the 1990s saw more refined definitions that posed governance as essentially different from international regimes following explicit agendas (Young 1989, 1997, 1999). Governance was specified as the sum of organizational rules and institutions (Donahue 2002, p. 1), the structure and ways of exercising power (Jasanoff and Martello 2004), and a system of rule (Rosenau and Czempiel 1992) both formal and informal at all levels of community (Rosenau 2003) beyond state government and its formal constitution. Governance encompassed informal structures, regulatory mechanisms, and inter-subjective meanings in the absence of formal authority. Rosenau and Czempiel (1992) highlight the *performative* character of governance. Governance happens where power is dispersed among proliferating actors (e.g., delegation of power to expert communities); where individual agendas are aggregated into new collectives (e.g., NGOs); where agreements emerge in self-regulatory and collective fashion (e.g., market rules and price mechanisms); and where effects are cumulative and often non-anticipated (e.g., migration movements).

To activate this rather passive understanding of governance as the collective mode of non-governing, Avant et al. (2010) focus on the proliferating range of agents, the “global governors”, on what they do and how they interact. They address the character of the relationships between actors to understand the “dynamics of authority” in global governance. Similarly, O’Neill (2009) highlights multi-stakeholder partnerships and global *economic* governance, focusing on the modes and sites of multilateral agreements, certification and trade schemes, financing and aid systems. Following Khagram (2006), O’Neill (2009) speaks of a “hybridized” global governance where “there is no one way in which global environmental governance occurs” (p. 21)—an observation much in line with what we here refer to as the great dispersal of global environmental governance. Similarly, Andonova et al. (2009) suggest a typology for studying transnational climate governance including three types of actors: public, private, and hybrid, which are networks of public and private.

Despite the conceptual development of GEG, the basic assumption of an underlying rationality, of self-conscious and intentional actors as well as of deliberative, interest-guided politics, has not fully disappeared in IR-guided scholarship. Moreover, the notion that global environmental governance is about pre-defined environmental problems or objects that precede the need for and the act of governance, keeps lingering. In this article, we tackle these assumptions by using STS-informed humanities and historical approaches. For our understanding of GEG, we borrow from the above-mentioned scholars the hybridized, dispersed, non-deliberate, aggregated, performative and often unanticipated constellations of actors, mechanisms, and effects. However, our historical analysis of GEG differs from classical global (environmental) governance scholarship on two key aspects: first, the exceptionalism of global environment as an object of governance; and second, the reciprocal relationship between environment and governance.

(1) *Environmental exceptionalism*: In global governance scholarship, the environment is often seen as an ideal case for studying how global governance functions, offering both empirical and theoretical insights. This is due to several recurring aspects of environment that appear in the literature. One key factor is the transboundary nature of environmental issues, which often involve shared resources, like the atmosphere and oceans or sea floors, and thus require collective action beyond national borders. Additionally, the complexity and interconnectedness of environmental challenges, which intersect with social, economic, and political factors, are said to frequently necessitate global expertise for effective policy development. Within this perspective lies the assumption that studying GEG can offer broader insights into global governance in general, implicitly placing environment alongside other major global concerns, such as global health, international trade and finance, human rights and so on.

While some insights from GEG may be transferrable to other areas of global governance, our historical analysis suggests that we must consider the environment as quite unique. On the one hand, the environment has been normalized into a cohesive policy object, a commercialized and neo-liberalized expert field through the proliferation and dispersal of actors, institutions and concepts on a global scale. Environment is ubiquitous today, environmental concerns are ever-present, permeating policy areas previously not considered ‘environmental’, from micro-, local- and planetary scales, to nearly every aspect of daily life. On the other hand, the spatial and temporal dimensions of the environment of planetary scale surpass the realm of the global. The planetary resists accessibility through common global governance mechanisms; it does not readily accommodate human concepts and agendas and global schemes (Chakrabarty 2019). Distinct from the globe, the planet refuses normalization—a condition that the concept of the Anthropocene denotes (Steffen et al. 2007).

(2) *The co-production of environment and governance*: To enhance our contemporary understanding of GEG, we address the reciprocity of governance structures and emerging environments. GEG, as we conceive it, involves the “co-production” of the global environment and its governance frameworks, reflecting a dynamic, evolving, and self-sustaining interrelationship between knowledge and political order (Jasanoff 2004). For instance, the 1972 UN Conference on the Human Environment (UNCHE) in Stockholm is often viewed as a starting point in the history of global environmental governance. However, it can also be seen as an early attempt to institutionalize the global environment—an endeavor made possible as “scientists made the global-scale environment visible” (Selcer 2018, p. 2). Thus, the UNCHE not only addressed global environmental issues but also symbolized the culmination of scientific and technological endeavors that made the idea of a ‘global environment’ governable. Consequently, the UNCHE represents both a *prerequisite* for and an *outcome* of early global environmental governance processes.

In summary, the question of the success or failure of GEG misses the point. The question of GEG’s effectiveness builds on the gross of governance literature’s problematic assumption that the environment is a governance field like any other. It falls short of acknowledging that the global environment in its planetary scales and boundaries is quite exclusively delegated to the authority and expertise of Earth System scientists to define the global environmental problems (Taylor and Buttel 1992). The normalized environment keeps refuting its normalization. GEG, so our claim in this article, has maintained this conundrum in a process we call ‘governing by proxies’, in a self-sustaining process of co-creating the mechanisms as well as the objects of environmental governance.

The historical process we are referring to extends beyond the more recent active use of the concept. The expression ’global environmental governance’ was not in much use before the year 2000 when it started to replace concepts such as ‘international environmental politics’ (Biermann 2004). Of the three constituent terms of GEG, the arguably new one was governance. The idea of governance went from relative insignificance to a central position in policy discourse in the 1980s, along with a trend toward what critics identified as market-based governance, sometimes perceived as gravely insufficient (Speth 2005), and which soon started to be called ‘depoliticization’ (Swyngedouw 2011; Bevir 2012). There was a search for alternatives to cumbersome state agreements and protocols (Kjaer 2004), and a growing sense of inefficiency and stagnation in environmental politics on the global scene. From the 1990s and onwards, the new language of governance, and GEG in particular, would be disseminated by both those opposed to and in favor of what the concepts entailed.

Key concepts such as ‘the environment’ and ‘climate change’ both have distinct careers, although with different temporalities. The former rising after WWII and leveling out from the 1990s. The latter insignificant until the early 1980s, since then rising rapidly almost to the same level as ‘the environment’. ‘Global environmental governance’, the full term, is hardly distinguishable (Google Books Ngram 2024). Web-based word searches are not the most precise empirical instrument (here limited to “books” in Google parlance, and to the English language), but in this case still convincing enough for our broad-brush comparison. Despite its modest circulation, GEG has been much discussed, tellingly though as much for what it is not as for what it actually is. GEG appears vague, somewhat hard to identify and often described as lacking. This characteristic of being everywhere and nowhere at the same time can seem puzzling given its assumed and desired existence for more than half a century.

Before Stockholm 1972, the idea of protecting the environment in a comprehensive world fashion was expressed in propositions of state-led initiatives, such as the never adopted idea of an International Environmental Government (Kennan 1970; Sohn 1973). Ways forward that had more immediate traction were international treaties, with the broad aim of conserving natural resources and governing transboundary environmental problems and conflicts. Such agreements drew on the tenets of classical liberalism, using legal mechanisms to create order between sovereign states and increasingly also encouraging environmental dimensions in international trade. As Stockholm, and later the Earth Summit in Rio de Janeiro in 1992, constructed a separate branch of international policy dedicated to the ‘environment’, it continued to draw heavily on classical liberal ideology. The rise of Neoliberalism in the 1980s and 1990s influenced environmental governance as it did other forms of governance. The components of the environment became managed through deregulation of state actors, self-regulation of an increasing number of private actors, and market principles (Mayrand 2022). By the time of the Earth Summit, commercial organizations were considered crucial partners for environmental action. This partnership was underpinned by the convergence in the years leading up to Rio of environmental and liberal economic norms that Bernstein (2000) calls liberal environmentalism. It could take the form of monetary contributions to government initiatives, or it could be through the self-regulation of businesses that increasingly equated environmental action to ’green capitalism’, appearing under multiple labels, such as “free-market environmentalism” (Anderson and Leal 1991) or “natural capitalism” (Hawken et al. 1999). Whichever form this governance took, the belief was that the mechanisms of the private sector were necessary for successful management of the natural world (Bergquist and David 2023; Biological Diversity Program, Peter Thatcher papers, 2023).

The concept of governance officially entered the realm of international environmental politics no later than the turn of the millennium during preparations for the United Nations World Summit on Sustainable Development in Johannesburg in 2002. During the preparations, the first Global Ministerial Environment Forum called on the delegates to “review the requirements for a greatly strengthened institutional structure for international environmental governance” (Engfeldt 2009, p. 226–227). The forum established a working group on International Environmental Governance, which was later supplemented by a working group on sustainable development governance chaired by Osita Anaedu of Nigeria and Sweden’s environment ambassador Lars-Göran Engfeldt. The latter recalls that “it felt natural to use the word ‘governance’ to encompass the broad spectrum of activities at all levels, including the non-state sector, involved in environment protection and sustainable development efforts. This notion was widely shared among negotiators and became henceforth the consensus before the Johannesburg Summit” (Engfeldt unpubl. 2023). This Summit is now widely seen as a watershed for private sector involvement, and hence of neoliberal principles, in global environmental governance.

A stylized timeline of GEG may start with the United Nations 1972 Stockholm conference and the establishment of The United Nations Environment Program, UNEP, the same year and run up to the present, citing major conferences and global agreements such as: The World Commission on Environment and Development (1987), The Intergovernmental Panel on Climate Change (1988), The United Nations Conference on Environment and Development (1992), The Kyoto Protocol (1997), The World Summit on Sustainable Development (2002), The 2030 Agenda for Sustainable Development (2015), The Paris Agreement (2015), and The UN Biodiversity Summit (2020). Already this (far from complete) sequence suggests that almost any number of more or less interacting institutions would not capture the phenomenon, nor certainly satisfy the perceived ideal. “Fragmentation” was therefore a term frequently used in the scientific literature of environmental international law and GEG in the first decade of the 2000s. Mostly lamenting the situation, reference was often made to the more than 1,000 existing international agreements, while anthropogenic activities, dispersed everywhere, remained the main driver of environmental and climate change (Zelli 2011; Isailovic et al. 2013). That some took a more sympathetic view of fragmentation (e.g., Acharya 2016) did not seem to change the overall understanding. Instead of assuming a coordinated governance architecture that did not so far materialize, we think it is more useful to approach it as a style of governance with a long history named ‘GEG’ at a particular point in time.

### From government to governance

That the environment we seek to govern emerges and is understood through the development of governance itself does not mean that GEG is an altogether unusual policy area. Quite the contrary. New processes of governance did in fact emerge. These involved: (a) institutionalization—be it through the establishment of national environmental agencies or through a multinational environmental fund such as the Global Environment Facility (GEF), (b) reconceptualization—for example through categories such as renewable energy or green-tech, and (c) the dispersion and proliferation of governance efforts. When this is the case, environment is *normalized* as a realm of policy. While this jars with our sense of ‘the environment ‘being an existential issue that transcends others, the practice of governance shows something different.

The more ubiquitous environmental demands became (recalling that the concept ‘the environment’ itself was invented to promote *integration*; Warde et al. 2018), the more they became standards to be judged *alongside* other standards in decision-making, like inequality or health. Arguably, in tension with environmentalism as a unique existential imperative, environment becomes part of a larger family of global governance objects. Its indicators can be compared with other policy tools like ‘literacy’. The idea of literacy as an individual capacity and metric was clearly an invention to grasp the skills involved in generating knowledge from texts, and then used to measure the success of education, but in turn improving scores of literacy (often measured by standard tests) became taken as the direct purpose and measure of being ‘educated’. Indicators thus indeed shape how knowledge is retained and emerges. Parallels can be found in environmental governance.

The early story of environmental governance is arguably simple. Before the late 1960s, there was very little that was *conceptualized* as environmental governance, and relative to what later developed, not very much environmental knowledge. When appeals were made about the ‘plundered planet’, the ‘limits of the earth’, or the ‘crowded planet’ (Osborn 1948, 1953, 1962), politics took the form of appealing to the educated cosmopolitan elite (and the American middle class) to be more ‘conservation minded’. Environmental knowledge was considered apolitical, something that was supported by international agencies and could be collated by collaborations of nation states regardless of political or social systems (IUCN 1950). As the ’age of ecology’ dawned around 1970 (Worster 1977; Radkau 2014), however, the scope of environmental concerns shifted and enlarged. This led to a period of rapid domestic institution building and to a new realm of environmental law and an expanding mobilization of expertise (Schleper 2019). Novel institutions like the US Environmental Protection Agency (1970) established under the Nixon administration in turn created demand for, and measurement of, a host of new phenomena, such as tolerable toxicity levels, indicator species, and so on that were packaged into larger political programmes (MacDonald 2011).

The new environmental agencies and ministries had limited remits beyond the borders of the nation state. The problem of environmental *governance*, acting beyond the standard although expanding remit of government, arose in part from the fact that governing an environment scaled up to whole-earth dimensions did not fit comfortably in a national framework (this includes those aspects that were *sub-national* but related to global problems.) Despite the national-level implementation schemes that were suggested in the three conventions that came out of the 1992 Rio Conference (The Convention on Biological Diversity (CBD), The United Nations Framework Convention on Climate Change (UNFCCC), The United Nations Convention to Combat Desertification (UNCCD)), there was no overarching institution or power which could compel nations to act. Another significant example was the Kyoto Protocol (1997) that was never ratified by the most major emitter, the United States. The ensuing international frameworks, however, were more than simply an *absence* of global government*.* This view would ignore the promethean effects of the ‘emergent environment’ in politics, across many scales. The generation of new governable environmental objects also created new possibilities through which behaviors could be related to environmental change through ideas like a ‘footprint’, and the extension of ‘carrying capacity’ and in turn governed. These possibilities went far beyond the regulatory staple of national or indeed international law, even though that remained important.

Simultaneous with shifting environmental concerns creating new avenues and imperatives for governance, ‘the environment’ itself came to be conceptualized through indicators that were thought to be amenable to be governed. The means of rendering the environment ‘legible’ (Scott 1998) were also those through which its transformation was enacted. Global environmental governance is the sum of those concerted, sometimes deliberate, sometimes unintended effects. The prevention of extinction, for example, was promoted through the invention of ‘biodiversity’ as a desideratum for policy (Wilson 1992; Rio 1992). This profound *reconceptualization* of environmental concerns assisted in the protection of particular species, but also made those species relevant above all *as a part of a diverse whole*.

Yet we argue for an apparent paradox. While ‘the environment’ was itself an increasingly integrative concept, often first articulated as being under threat all around the planet, the importance of the ‘global’ scale of environmental issues did not create a concentration, but rather a *dispersion* of environmental governance. As the scope of governable objects ran far beyond the particular remit of environment agencies, *everything* was, potentially, mobilized as environmental. Everyone could in principle become an agent of this process: governments, NGOs, consumers, firms, municipal authorities, universities, even militaries (Biermann and Siebenhüner 2009; Waterman et al. 2004; on environmental security, e.g., Schulz 2010), and sometimes those diverse agents united in complex ‘green networks’ for example managing various types of environmental certification. In turn, the action of each of these ramified down long supply chains and impacted places far away.

The actors were also dispersed because the ‘object’ itself was highly dispersed; the governance of the environment is now often found on a continuum expressed in an index rather than simply, and discretely the protection of a valued site. One does not only ‘conserve’ a particular place as in older forms of conservation based on National Parks and other protected areas, for example. Networked nature made every place (still perhaps some more than others) a contribution to biodiversity, or carbon emissions, or the nitrogen cycle, in ways that only became relevant when considered on a much larger, possibly global scale. Opting out of global environmental governance was not possible because the governable environmental objects were not contained. GEG worked through the *proliferation* of objects. It was comprised of practices exercised without being necessarily able to identify the set of intentional actors nor to ascertain an immediate certain effect.

Unable to rely on a single authority or sovereign power, GEG was *multi-agential* and institutional and relied on *intermediate* actions, tools, and proxies. Some agents engaged with or increased their engagement with the environment exactly because of the lack of reliable authority. For example, the non-profit organization Climate Group, which seeks to bring together sub-national governments and large businesses to fight climate change, was formed specifically as a reaction to the United States’ decision not to ratify the Kyoto Protocol (Hoffmann 2011). Alliances of cities for environment and/or climate action have formed in many countries and regions. There are even global networks such as the 2500-member Local Governments for Sustainability, founded in New York in 1990 (ICLEI 2023), and international networks of national alliances, such as Alliances for Climate Action (ACA 2023). Checks and balances are important. GEG worked not just through regulations and restrictions but also through incentives and benefits. Introducing these became a prolific activity on all scales from the local to the global: polluter pays principle, encroachment regulations, carbon budgets, standards, thresholds, tariffs, tax reliefs, Clean Development Mechanism, emission certificates, etc., but with less global coordination than early proponents of GEG had wished for.

Emergent governable objects and associated forms of governance also generated a *recategorization* of established forms of governance where practices that were previously not framed in terms of the environment became promoted as environmental. This is evident in relation to renewable energy. For example, at the beginning of the twentieth century, wind turbines were first constructed as a way of electrifying and empowering rural areas. In the context of the oil crisis in the 1970s and 1980s, wind energy was seen as an alternative to both fossil and nuclear energy (Nissen and Christensen 2009). In the twenty-first century, wind energy became a vital element of ‘green development’, explicitly linked to carbon budgets and sustainable development goals.

Most environmental policy is local and project-centered. Do governments put planning limits or pollution controls on particular enterprises or sectors? Do development banks loan money to encourage a particular kind of activity? What is the most ‘environmentally-friendly’ purchase, and how do consumers know? This kind of global governance is not distant and abstract; on the contrary, it is concrete and intimate. GEG and its set of indicators and tools of assessment permit a means by which all these projects and choices can contribute to global governance, but also allow *all* projects to be so judged, whether the people promoting them like it or not. However, alongside the plethora of varied actors—as many governors as there are people, plus the organizations to which they belong—the means to monitor the projects and assess their value are often extremely limited. Often, they are provided by the initiator of the project themselves (a vendor of carbon credits, or manufacturer of ‘greener’ cars). To tackle issues of trust, new agents of governance such as the Forest Stewardship Council (FSC) and the Global Reporting Initiative (GRI) emerged to provide ‘accreditation’ and ‘transparency’. The mechanism works even *within* large organizations: BP and Shell found it easier to create an internal market for carbon among their own projects rather than regulate them from the top.

Once all policies and behaviors become assessed according to their environmental effects (still perhaps an ideal rather than a reality), we are left with the need to make trade-offs between ‘environmental’ and ‘non-environmental’ rationalities, but also *between environmental priorities*: is nuclear power, for example, anti-environmental (risk of radioactive contamination) or environmental (reduces carbon emissions)? GEG is the means by which projects are linked to a global environment, but also the means to contest and negotiate those goals and claims. GEG is not simply mainstreamed environmental governance, but integrates dissonance, disbelief and denial, as the long history of profound disagreement in COP meetings has demonstrated along with controversy over the IPCC (Hulme 2009; Miller 2022).

### The environment as an emergent concept

The governable global environment emerged in particular forms and formats. Glaciers, forests, species, soil, oceans, and atmospheres have been studied for centuries and for various reasons. They were not always ‘environmental’; they became environmental as they were summoned into the global environment as a systemic entity. They were construed as ‘governable objects’ and in this course, we argue, they changed quality. The air did not cease to be the air but also became a place of growing ozone accumulation and a sink of emitted atmospheric carbon. The oceans moved beyond being solely a resource pool and home for fish stocks, and also became seen as a carbon sink, a system of water circulation, a layered system of trophic zones, as well as a source of rising sea levels, while the sea floor became understood as a repository of sediments that spoke of deep time climate change, ultimately digitally assembled (Helmreich 2011; Isberg 2023; Wickberg et al. 2024).

The global environment was assembled from such governable environmental objects and their relations, transformed in international science, assessments, institutions, negotiations, and agreements. This process of assembly suggests an alternative historiography to the one in which environmental issues are discovered by science and later handled by politics (Latour 1993). Instead, we identify the environment as being both the object *and the effect* of global environmental governance; both shaping and being shaped by how institutional frameworks and responses developed throughout the postwar era. The environment emerged conceptually and materially through particular practices and ways of seeing, measuring, and relating to the world. Crucial to the emerging environment was the idea that it should be guarded, protected, or governed by humans, despite often having been severely affected by humans.

The first fledgling understanding of the global environment coincided with reconstruction after World War II. Incidentally, and importantly, the world in which it grew up was marked by the shaping of a new global architecture of institutions for peace keeping, trade, social, educational and cultural development, many of them branches of the United Nations. Other institutions were shaped for collaboration on science (ICSU), meteorology (WMO), agriculture (FAO), the Bretton Woods institutions for financial stability and economic development with the IMF and the World Bank, and for some countries the OECD, which for a moment in the early 1970s were caught by the winds of environmental change (Schmelzer 2012). For nature proper there was the International Union for the Conservation of Nature (IUCN). These were species in a rapidly growing postwar ecosystem of globalizing acronyms, signaling the acknowledged need of international cooperation.

They shared and still share many properties. It is possible, though, to identify some main features of these institutions that can explain the exceptionalism of the environment. First of all, they were primarily based on states and state interests, and states could find their roles in the postwar institutional architecture in most areas. Secondly, the institutions had fairly identifiable and distinct criteria of success and hence transparent roles and work packages for states to take on, either as providers or as beneficiaries. Contributions could be measured in dollars and loan giving and conflict resolution, or for that matter modernization of the developing world could be assigned metrics of economic growth or the scale of loans.

‘Environment’ was far less easy to grasp as a policy challenge. It emerged as a blanket concept of concern for a planet Earth that seemed increasingly vulnerable, shrinking, overpopulated, overexploited, polluted and under-protected (Vogt 1948 and Osborn 1948 are seminal texts). This perception was novel, despite eruptions of previous concern over deforestation, flooding, drought, extinctions of individual species, and soil decline (e.g., McNeill 2001; Barrow 2009; Dotterweich 2013; Warde 2018). Yet it wasn’t immediately obvious what the appropriate environment was actually like, or what share of the responsibility was carried by a certain country, besides the obvious fact that it carried the prime responsibility for its own territory. The “global” in global environmental governance was, in other words, not a given, it had to be negotiated, sometimes involving new actors and organizations. The threat to the environment was to be found everywhere, but this could be viewed as arising from the ubiquity of population pressures or resource scarcity or local polluters. Environmental NGOs like Greenpeace and the World Wildlife Fund (WWF) appeared next to the governmental, academic and economic institutions, to point to the limitations of the national level to address the global.

Establishing the scales of the environment, as Perrin Selcer (2018) and Etienne Benson (2020) have shown, depended on the institutions and actors with epistemic authority as well as practices and technologies of measuring environmental phenomena. Establishing the spatial perimeters of an environment is a self-evident part of any kind of environmental knowledge production, from a small forest to the entire planet seen from outer space. It took time for environmental sciences, a term itself only coined at the end of the 1950s (Honeybun-Arnolda 2024) to build up a knowledge base, and often the development of that knowledge base (such as localized air pollution or water quality) emerged in response to the concern of local authorities over health and specific hazards such as pesticides (Carson 1962). On a more international and global scale, the threat to the environment was presented as a function of phenomena for which data was available at a large scale: population from censuses, and resource exhaustion from demand and estimates of future stocks.

But the scales of the environment are also a matter of time. In comparison to other, non-environmental governable objects—be it unemployment or literacy rates—the temporalities of the environment operate on vastly different registers and encompass phenomena beyond the sociopolitical domain. Questions of whether environmental issues unfold slowly or rapidly, evenly or unevenly, and whether they constitute an immediate crisis or a potential future problem, are at the very core of how said issues should be addressed politically and understood scientifically. Time, for example, was the essence of biodiversity governance. The interdisciplinary approaches that fed discussions about global extinction rates encompassed natural and social sciences that went far beyond the boundaries of human history. Critics of ‘mass extinction’ argued that species loss was a natural phenomenon, but historical biogeographical, paleo-botany, and geological analysis were used to argue that the current rate of vanishing species was potentially several hundred times higher than ‘normal’, solely due to human impact. It was the process of mapping human history into planetary time that redefined biological diversity as something in crisis and in need of management (Myers 1979; Ehrlich 1982; Sepkoski 2020). GEG has in this sense “synchronized” (Jordheim 2014) human and nonhuman temporalities in particular ways that have shaped the temporal properties, and sense of urgency, of the global environment (Sörlin 2022).

The history of GEG must therefore be seen as emerging in tandem with new ways of measuring and knowing environmental phenomena, for which the institutionalization of new environmental sciences were central. Exploring how environmental governance is not just a response to material changes in the environment, but also the producer of particular ways of making environments (Sörlin and Wormbs 2018), opens up a new way of understanding the history of GEG.

## A new history of GEG 

The environment of 1972 was not the same as the environment of 1992 and even less of 2022. This is not just because of the obvious fact that the Earth is a dynamic and ever-changing place. It also reflects profound changes in how people conceptualize the environment and hence act in relation to it. What they think they act on is not a stable entity, and the very creation of new modes of governance changes what they think they are governing. Hence, to ask whether the initiatives of the early 1970s, for example, were a success, is to some degree to miss the point. If we answer the question from the perspective of today, we are not using the same criteria of success. The changes that have come at this time have not been the only moments of revelation in modern environmental thinking. For example, many early environmentalists speak of how they were inspired by reading Rachel Carson’s *Silent Spring,* first published in 1962. The power of that book was not in revealing how something already loved was being damaged; it lay, to a degree, in laying out how something that people did not even know existed, the evolving ecosystem in which they lived, was already deeply imperiled (Warde et al. 2018, p. 6).

The Carson moment not only revealed a new form of environment, but also led to a redefinition of what counted as environmental components. Conservation, once focused on preserving singular landscapes and species, developed into a more integrated activity that incorporated the interactions between all organisms as well as their physical surroundings. The identification of these ecological components increased in parallel with a growth in the number of taxonomists and significant steps in the sophistication of technologies used to classify species seen from the 1960s. By the 1980s—particularly following Terry Erwin’s hypothesis that there were an additional 30 million arthropod species more than had previously been estimated—identifying the number of species on earth, analyzing how they related to each other, and using this information to inform nature management became key themes in environmental thinking and governance. As the science developed into fields such as molecular biology, visions of the environment only expanded further (Erwin 1982; Costello et al. 2013).

These kinds of changes did not mean a wholesale flip in states, but the supplementing and eventual eclipse of a postwar style of thinking by something different. In the academic and campaigning literature of the early to mid-1970s, including that which framed the Stockholm conference, the environment had largely been imagined as a collection of local or regional biomes. These were under threat from growing human numbers, traced by the upward curve of population and the downward curves of resource stocks, disappearance of treasured places, soil erosion and looming scarcities. Although earlier thinkers had posited more integrative models of the ‘biosphere’ or global climate (see, e.g., Aronowsky 2018 on Vernadsky, Hutchinson, Lovelock; Fleming 2007 on Callendar) these were sketched roughly and had little policy traction. By the end of the 1980s, the policy landscape was different. This meant that the environment was also different. Climate modeling (and the associated temporalities and ‘budgets’ of oceans and atmospheres as carbon sinks) had entered policy (Lahn 2020) not just through global warming, but also the analysis of phenomena like nuclear winter, and the ozone holes (Dörries 2011; Grevsmühl and Briday 2023). Yet this was not just about natural science. It was also about geopolitical decisions made in those decades, and significant changes in economic governance and the social sciences.

We will now move to set out, briefly, three significant stories about these changes, that in some sense represent a bridge between two worlds—of 1972 (Stockholm) and 1992 (Rio). These should not be seen as aspects of a single story, or the necessary consequences of each other. Rather, they are three intertwined histories that all strongly conditioned processes of GEG over this period and up to the present. The first is the emergence of Earth System science; the second is the geopolitical parameters set by debates and agreements over national sovereignty, especially with regard to ‘North–South’ relations; and the third is the rise of new ideas in economics and governance loosely described by the term ‘neoliberalism’ (which is viewed both positively and negatively by different political constellations). 

### From IPAT to the earth system—and its proxies

The first significant story concerns the rise of the Earth System as a structuring logic of environmental sciences and governance. Source material from the 1970s shows a striking absence of any thinking of an Earth System, or indeed wider indicators of the state of forests, oceans, biota, etc. Pollution registered, but it was fundamentally treated as a local phenomenon, even if it could be distributed in many incidents across the planet, for example the way nuclear fallout was shown to do in the striking visuals from atomic bomb tests in the Pacific (Machta et al. 1956). The problem of sustainability at this time was conceived as one of resources, partly related to population, but above all to energy as in early conceptions of ‘The Sustainable Society’ (Pirages 1977). This was expressed at the time in what is now sometimes called the Commoner-Ehrlich-Holdren or ‘IPAT’ equation, where IPAT stands for Impact = Population * Affluence * Technology. In other words, impact was viewed at the largest scale in a comparatively simple and functional way. Energy was seen as fundamental by the emerging field of ecological economics at this time (Kenneth Boulding, Herman Daly, Nicholas Georgescu-Roegen) because of the problem of entropy. It was developed in an abstracted format as these scholars perceived society moving beyond the capacity of the current energetic system. This was conceived as a problem on a very grand scale (Meadows et al. 1972; Catton 1980) but it was not connected to the Earth System.

We suggest that the 1980s saw a major shift in which ‘the environment’, a paradigm focused on ‘limits to growth’ largely via resource exhaustion and pollution—emerged reconfigured as something concretized and institutionalized. This reimagination of the environment included many new sources of data connected to the political and economic realms. Even though the notion that the Earth is an interconnected and complex system had been expressed already by Vladimir Vernadsky in his 1926 book *La Biosphère* (in Russian, in French in 1929) and had gained traction since the 1960s—especially due to the rise of systems theory and increasing political attention to the environment—it did not have a coherent research program attached to it. Between Stockholm and Rio, the Earth System took form gradually, with the most significant developments unfolding in the 1980s. It gained spatial and temporal properties, assembled data, institutions, computational capacity and scientific networks, and seized epistemic authority as the main interpretive framework for knowing and predicting the planetary environment.

Precursors to what would become Earth System Science existed already in the years preceding the Stockholm conference. In the 1960s, the ecologist G. Evelyn Hutchinson, who had started working on Vernadsky’s ideas already around 1940 (Pireddu 2023) drew together the major cycles of the biosphere as a unified system, from water and energy to carbon, nitrogen, and mineral cycles (Hutchinson 1970; Aronowsky 2018). The Odum brothers envisioned the Earth as a synthesized energy and social system starting in the 1950s leading to the beginnings of systems ecology (Hagen 1992). In the first half of the 1970s, James E. Lovelock and Lynn Margulis (1974) proposed their Gaia theory. It sought to conceptualize the biosphere as a single living entity, but lacked the empirical and conceptual underpinnings. Modeling capacities were increasing too: throughout the 1970s, large-scale data-oriented research programs such as GARP (Global Atmospheric Research Program) and the development of more advanced computerized climate models, General Circulation Models (GCMs) enabled increased opportunities to encompass the entire planet in a modeled format.

Yet, it was only in the 1980s, with the establishment of NASA’s Earth System Science Committee (ESSC), who coined the very term Earth System Science, that the notion of the Earth System gained institutional and epistemological momentum. Francis Bretherton, the chairman of the ESSC gave a revealing answer regarding the novelty of Earth System Science in an interview with *Science* in 1986: ‘Many of the observations we need are already being made for other reasons, such as weather forecasting’. He stressed that the main difference to previous approaches was conceptual rather than technological: ‘It’s more an attitude of mind. We want to make sure that we go the extra mile—that we cover everything.’ This idea of modeling ‘everything’ and not just a particular environmental object—such as the atmosphere or ocean currents—did not have scientific traction around the Stockholm conference, but was the self-evident underpinning of the Rio conference 20 years later. In practice, however, Earth System Science relies on proxies for the larger systems it aims at modeling, for example using ice cores as representatives for the past climate (Isberg 2023). In this way, while Earth System Science still is confined to a partial and situated picture of the planet, its ultimate goal is to present ‘a view from nowhere’ (Haraway 1991).

This epistemic shift brought new planetary environmental dimensions into view. Placing the genealogies of a *systemic* earthly environment in the foreground revealed a new range of knowledge formations. Among others, the (re-)emergence of the biosphere as a scientific object in the 1960s and its development into the Earth System, as well as subsequently developed concepts like Sustainable Development (WCED 1987), conceptual frameworks like the planetary boundaries (Rockström et al. 2009), and institutional settings like the International Geosphere-Biosphere Programme (IGBP), the IPCC, the United Nations Framework Convention on Climate Change (UNFCCC), and the Convention on Biological Diversity (CBD). Together, these concepts, frameworks and policy arenas created new categories for governance. They also spurred new forms of ecological management that did not seem to exist at all in the 1970s.

The Earth System introduced novel temporal and spatial scales as well as novel complexities to the understanding of the environment and its governance. For the understanding of environmental time, the Earth System worked as a large synchronization device, both figuratively and literally. It incorporated scientifically informed timescales—past, present, and future—into a new temporal framework. Rather than environmental deterioration reflecting a linear and reversible trajectory of change, the deep past and the future of the Earth became entangled with the present. On a spatial scale, the Earth System privileged global processes over local variations and channeled concerns over deforestation, flooding, drought, and soil decline into a complex planetary machinery. The Bretherton Diagram, produced by the NASA Earth System Science Committee in 1986 and named after its chairman, is one representation of the birth of the Earth System as an object of knowledge and as an object of governance (Barton 2020)*.* The insertion of ‘human activity’ into the diagram, put humanity—as one unified category—on par with geochemical and geophysical dynamics and revealed how anthropogenic effects were now an integrative feature of the entire Earth System (Steffen et al. 2020).

Consequently, the organizational ‘earth-turn’ brought actors and institutions onto a global arena which was not commonly considered ‘environmental’, or part of an institutionalist history of GEG. NASA for instance, with its ‘Mission to Earth’ program, became an Earth Agency; it shaped and produced ideas of what the (global) environment is and how it should be governed. The Earth System claimed to represent the planet as a whole, unifying its physical features, elements and cycles, and eventually included human, cultural and social exchange systems as well. It was necessarily an abstraction and a simplification, which partly explains its success, but also its tendency to homogenize geographical and political inequalities (Coen and Albritton Jonsson 2022). Different from previous local, regional and national environmental concerns, the Earth System had to be governed by proxies standing in for certain features and categories (Mulvin 2021). Examples are ice cores standing in for global climate history, sediment cores as proxies for Earth’s development, or entire earth entities like the world ocean, or global average atmospheric temperature standing in for climate change (Paglia and Isberg 2022). These Earth System categories started to appear as justifications for policy, or even as objects of policy. The Earth System scale produced new kinds of governable objects: it enabled a governing-by-proxy relation between local environments and global environmental governance.

### Sovereignty and the distribution of responsibility

Our article began with the lament of Swedish diplomat Sverker Åström about the necessity but probable failure of what we might now call GEG, or at least one version of it. However, Maurice Strong, the highly energetic and influential Secretary General of both the 1972 Stockholm Conference and 1992 Rio Earth Summit, had a more sanguine if somewhat cynical outlook on sovereignty:

It is only when nations find themselves incapable of exercising their sovereignty effectively or advantageously on a unilateral basis that they will agree—reluctantly—to exercise it collectively by agreement with other nations. It is seldom that nations enter into arrangements which restrict their ability to exercise their sovereignty until circumstances compel them to do so (Strong 1973, p. 706).

During the preparation process for the Stockholm Conference it was for the most part countries of the global South, concerned over possible limits to economic development, that were adamant about safeguarding their sovereignty over natural resources and national environmental policies. This represented an assertion of (what for many of those countries was) newly gained national independence and a break from their colonial past, a period during which resource exploitation was primarily in the hands of external (often Western) powers. Brazil in particular was highly critical of industrialized countries for allegedly using the environment as a pretense for imposing what some development economists and political leaders in the global South saw as incipient Limits to Growth—a sentiment reinforced by the Club of Rome report of that name published in the months before the Stockholm Conference. Principle 21 of the Stockholm Declaration asserted, crucially, ‘States have… the sovereign right to exploit their own resources’ (UN 1972).

Such battles were also fought in other arenas: the G77’s proposal for a ‘New International Economic Order’ in the 1970s, which would serve to regulate international markets for raw materials and improve terms of trade for the Global South, was rejected by most Western nations (Bhagwati 1977; Humanity 2015). Two decades later at the 1992 Earth Summit, debates on biodiversity, encompassing genetic resources and thus the raw material for biotechnology, became central to debates about sovereignty. In the process of creating the Convention on Biological Diversity, the biodiverse-rich nations of the Global South worked toward a framework that could create contractual arrangements between providers and users of genetic resources, allowing them a share of any financial benefits. These attempts were several times blocked by countries of the Global North who had long benefited from exploiting the genetic resources of the South and were reluctant to discard the existing agreements on intellectual property rights (Macekura 2015).

At the Earth Summit, sovereignty concerns had also come to the surface in some countries of the global North. Most prominently, the USA whose president George Bush, in discussions over the framework convention on climate change that was a centerpiece of the Rio summit, declared that “the American way of life is not up for negotiations. Period.” (Hudson 2018). Future American presidents and legislatures would refuse to ratify and even renounce international climate conventions.

Further demonstrating the enduring influence of the principle of sovereignty over international environment politics, the legally binding Kyoto Protocol of the UN Framework Convention on Climate Change would prove short-lived. After the end of its first mandate period lapsed at the end of 2012, the top-down Kyoto approach was replaced by the 2015 Paris Agreement’s policy concept of Nationally Determined Contributions (NDCs). As the name implies, NDCs are a bottom-up approach to climate governance underpinned by the principle of national sovereignty, with each country independently deciding upon the amount of carbon emission restrictions they will “contribute” to the global effort of combating climate change.

Sverker Åström had wistfully evoked “world government” in his 1972 *Ambio* article, knowing, as a veteran if idealistic diplomat, that such an institution would never come to pass, at least in his lifetime. The global governance that has evolved consists of a far more spatially and sectorally dispersed set of institutions that encompasses, in the realm of environment, a wide array of treaties and agreements, laws and legal principles, organizations and authorities, recurring conferences and international processes, and scientific concepts and indicators that serve as the basis for policy and for assessing progress. We might also include the behavior of firms, private individuals as property owners or consumers, non-governmental organizations, sub-national governments as well as networks composed of several of those. The dispersal of governance takes place at different levels, and each entity is supposed to contribute in their own way to the greater good of the global environment. Often such actors are invited to participate in more formal processes, such as Environmental Impact Assessments and consultations.

This dispersal partly represents wider trends in governmentality and the ‘little tools’ of governance (Asdal 2015; Asdal and Huse 2023). In the case of GEG, they are also a way of negotiating around the bounds imposed by sovereignty. The institutional infrastructure of the international system (particularly exemplified by the United Nations architecture) being undergirded by sovereignty presents a significant constraint on effectful dispersion of environmental governance, encouraging the emergence of new forms. These include an increased emphasis on municipal and transnational initiatives, notable in the ‘Local Agenda 21’ of the Rio summit, which also sought to draw ‘alternative voices’ that had protested outside of the Stockholm conference into the debating halls in 1992—even if the final agreement remained in the hands of nation states. When the head of UNEP, Mostafa Tolba, felt energy draining out of UN efforts to develop environmental policy in the early 1980s, he turned to business organizations such as the International Chamber of Commerce for support. This contributed to the gradual institutionalization of voluntary systems of governance and certification, and increased the popularity of the idea of ‘sustainable development’ (Bergquist and David 2023). This paradigm has become dominant in GEG and is today present in diverse contexts from the sustainable development goals (SDGs) to corporate mission statements. This led, one might argue, to rhetorical cohesion but practical dispersion in governance. Businesses similarly resisted having rules and regulations imposed on them from above and instead, they also took up the model of delivering ‘pledges’ by which their environmental efforts could be judged, just like nation-states that would not cede sovereignty.

What then were the mechanisms of enforcement in this dispersed world of sovereign actors that could range from the sovereign state to the sovereign consumer? How was accountability to be generated? There is no central authority that can really hold agencies or nations to account for how they are impacting the global environment, or even a central authority that gets to define what the ‘global environment’ fully entails. Governance operates through information: ranking, monitoring of pledges and targets, and by the (alleged) threat of consumer power, or simple embarrassment.

### Neoliberalism, and the market as governance

Neoliberalism had much influence on the dispersed form that GEG eventually came to take. The 1970s could be regarded as a decade of not just crisis (Ferguson et al. 2010) but possibilities. The parameters of emerging global issues, including the environment, were still under negotiation. The fallout of the oil crisis also remained to be seen. In these respects, and others, the 1980s was arguably a decade of foreclosure. The eighties saw a marked turn to neoliberalism, a widespread debt crisis among developing countries, a wave of international protests against World Bank austerity policies and projects it funded that were seen to be environmentally and socially destructive, and the crystallization of the sustainable development paradigm.

The ‘neoliberal turn’ of the 1980s was in part characterized by a politically rightward shift in key Western governments. Ronald Reagan became president of the USA in 1981, Margaret Thatcher became prime minister of Britain in 1979, and Helmut Kohl became chancellor of West Germany in 1982. The turn reflected a shifting economic regime that favored growth, globalization, privatization, and free market principles. While this development built on a longer historical trajectory of liberal internationalism and visions of global governance, it gained particular momentum in the early 1980s (Ikenberry 2009; Martin 2022). This economic regime is often associated with politically forthright economists such as Friedrich Hayek and Milton Friedman who saw the role of global economic institutions primarily as guardians of the market against potential threats posed by democracy and the nation states (Slobodian 2018).

In the case of major international financial institutions like the World Bank, this was represented by the mechanism of structural adjustment lending, whereby loans were provided to developing countries on the condition that the borrowing country would enact specific reforms in their domestic economic policy. These adjustments often included stipulations that involved relinquishing previously nationalized services (such as water, electricity, telecommunications, or manufacturing) to the private sector, reducing trade barriers (although often argued for by countries in the Global South), and boosting production for export rather than domestic needs. Peet (2009/2023) argues that the International Monetary Fund, the World Bank, and the World Trade Organization, advanced a hegemonic order as ‘global governance institutions’ in which a centralized (quasi-state but unelected) body of experts shared a common ideology. The principle of free trade in certain cases undermined national environmental legislation, for example when the WTO deemed environmental legislation to be distorting competition (O’Neill 2017). In this way, neoliberalism has contributed to the dispersion of GEG by encouraging a move away from legislation and toward voluntary schemes.

These changes also reflected important shifts within the practice of economics, also related to its increased use of mathematical models of optimization. Proofs were delivered against the possibility of planners being able to deliver policies that optimized outcomes for all market participants. Microeconomics, focusing on individual decision-making, gained prestige over the apparent failures of Keynesian macroeconomics. ‘Rational expectations theory’, particularly associated with the work of Robert Lucas, argued that ‘the wisdom of crowds’, that is, the disaggregated behavior of individual consumers guessing outcomes, produced the most optimal result and could not be captured by self-interested groups (e.g., Lucas 1972). This enhanced prestige of market solutions within economics gave intellectual underpinning to politicians suspicious of or opposed to planning, and led to a new mission for governors: artificially creating markets for ‘products’ and ‘services’ which had not previously existed, to optimize the production of such ‘services’. From the 1960s onwards, economists also became more confident in valuing non-market services (such as environmental damage or ecosystem services), allowing them to be incorporated into cost–benefit analysis and the design of new markets (Banzhaf 2023).

Neoliberal governance thus involved creating ’neoliberal natures’: turning resources into commodities rather than guaranteed ‘necessities’ or assets for ‘security’. The economic potential of these commodities as they flowed through international capital markets was laid out in the Brundtland Report of 1987. By legitimizing the idea that economics and ecology worked together for the benefit of humankind, neoliberal thought also began to shape practical conservation, and projects were increasingly reconfigured according to market dynamics (WCED 1987; Büscher 2012). Whether water, forest lands, or mineral reserves, the idea was that market forces would allocate these resources most efficiently, ostensibly replacing the need for state-based regulation (Myers 1988). From a more extreme neoliberal viewpoint, environmental problems were simply problems of ownership and marketization. If for example, ownership of a dwindling fish species was clearly defined, then market forces would through mechanisms of supply and demand ensure that the species will never go extinct (Dryzek 2013). The broader global environment became redefined as a set of ‘ecosystem services’ whose value can be measured or aggregated (Daily 1997; Costanza et al. 1997; critiques in Norgaard 2010; Ernstson and Sörlin 2013), although this idea has had more influence rhetorically than in practice.

As such, markets do not always spontaneously appear. Although business has also sought to create them, neoliberalization can be regarded as a form of *re*regulation as much as *de*regulation. States’ powers should not be considered to have reduced as much as they have been redeployed to ‘transform previously untradable things into tradable commodities’ (Igoe and Brockington 2007, p. 437). While pure neoliberal environmental governance has been relatively limited in scale, neoliberal thinking has influenced GEG in many ways (Dryzek 2013). In the case of conservation, neoliberalism might be exemplified by the creation of protected areas as business ventures (e.g., ecotourism). The new governance instruments associated are many: carbon budgets and taxes, the ‘right to pollute’, green certifications and many more. It should be noted, however, that many of these policies are not entirely new. Tradable licenses to pollute and forms of ‘polluter pays’ taxation were long-term staples of environmental economics from the 1960s, and are to be found as suggestions among economists such as John Krutilla, John Dales, Thomas Crocker and Herman Daly (Pirages 1977; Banzhaf 2023).

### Recent pathways

Our story so far—itself purposely and congenially dispersed—indicates that global environmental governance has evolved in much broader strands of activities and in a far less coordinated way than the conventional, linear GEG narrative suggests. States, global institutions and numerous other, also very local, actors around the world have transformed their encounters with grand challenges of environment and climate in multifarious and complex ways. The structured effort of actors, especially states, that was assumed when global environmental governance was projected and prescribed in policy circles from the late 1960s is only a part of this development. GEG is, we posit, so far better understood as a more open-ended historical process that in many ways belies the notion of a progressive move toward an ever-growing coordination.

This is not to deny that much of this open-ended process was predicated on proactive environmental initiatives and policies in the past, often a direct result of ambitious governance applying rigorous methods and tools. But the actual history of GEG is a much larger, and far more interesting story. We do not know where future global environmental governance will go. However, we do think it is useful to read some of the more seminal recent governance initiatives against this long experience of the past. Not to suggest or privilege any particular initiative, but rather as an attempt to deepen understanding in the face of large uncertainties and a future that remains open. We have identified a set of broad governance pathways that have shaped and are still shaping global environmental governance as Agenda 2030, now past midway, moves on. As we will see, they align to a large degree with the analytical GEG trajectories that we have presented in the previous sections, although it should be said that we do not suggest that they overlap in any complete or exclusive way; governance domains tend to defy sharp, long-term definitions, which is part of the logic of dispersion. At the end, by way of conclusion, we will sketch some contours of a theoretical and meta-historical framework that may bring some cohesion to what is soon a century of dispersal, which largely remains to explore and make sense of as a part of modern history.

**Fig. 2:**
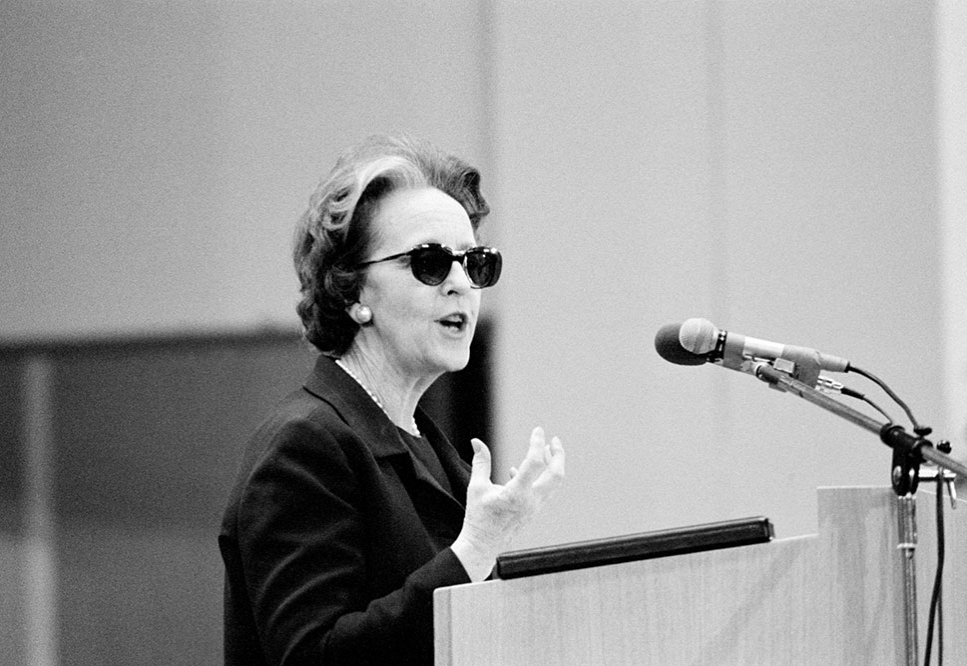
Economist Barbara Ward speaking during the Stockholm UN 1972 conference

*Pathway 1: Targetization.* Global environmental governance since the late 1990s has been increasingly goal setting. To a significant degree this has been made possible precisely through the development of metrics, indicators, and proxies that emerged as “governance by proxy” described above in section about the significance of Earth System Science. Steering with (relative) ease to set goals rather than hard-to-decide rules was also a core element of the neoliberal soft governance. Targets were meant to legitimize and integrate more local initiatives and thus encourage action below the international level. The Millennium Ecosystem Assessment, concluded in 2006, was about goals. The 17 UN SDG goals, established in 2015, are under evaluation as the Agenda 2030 decade progresses. However, this soft governance is also characterized by the fact that no one has a clear responsibility for meeting the goals and avoids binding international agreements. Results so far are disappointing. Virtually none of the SDGs meet midway expectations and those for environment and climate are far from being reached (IPCC 2022; UN 2023). In the words of UN Secretary General António Guterres (2023), ‘Unless we act now, the 2030 Agenda could become an epitaph for a world that might have been’ (Foreword, on p. 2). Taking the broader picture of major goal setting efforts since 1972, the results are even more alarming (SEI and CEEW 2022, on p. 34). The lack of progress has caused observers to question the intense ‘targetization’ (Pickering and Persson 2020; Persson et al. 2022). One could even say that constantly setting new targets serves procrastination and inaction. Nonetheless, these global targets have legitimized numerous local initiatives. Where targets are given without clear implementation frameworks, organizations have often taken the initiative to create their own principles for action that are both highly localized and internationally applicable (Fig. 2).

*Pathway 2: Justice/equity.* An equity dimension was always linked to environmental and climate governance. It was built into the background documents of the Stockholm conference (Ward and Dubos 1972), and during the conference and repeatedly since then it has been manifested in a tension between the global North and South. Principle 11 of the Stockholm Declaration stated that all states’ environmental policies ‘should enhance and not adversely affect the present or future development of potential of developing countries’ or ‘hamper… better living conditions for all’ (UN, 1972). It still plays a major role, with an added and growing Anthropocene component depending on income, lifestyle and technological level of existence that determine the level of anthropogenic impact. The Planetary Boundaries framework has increasingly addressed these issues (Steffen et al 2015a, b; Rockström et al. 2023) and has also been suggested as a platform for new ideas of governance based on Earth System boundaries (Albritton Jonsson and Wennerlind 2023). Barbara Ward, the British economist (Fig. 2), had developed similar ideas in her book *Spaceship Earth* (Ward 1966, 1982; Höhler 2015). Ward also drafted the 1974 Cocoyoc Declaration, where she speaks of inner limits of social justice, and outer limits where systemic boundaries were threatened. Following Ward’s initial synthesis, Oxford economist Kate Raworth (2012, 2017) introduced doughnut economics as another bounding metaphor, with an inner bound representing the social foundation for a safe and just space for humanity and an outer bound of the doughnut representing the ecological ceiling. The increasingly strong link between environmental and equity thinking has been a significant catalyst for the dispersal of GEG into other realms. All in all, the *justice/equity* aspect relates to the North–South divide that goes all the way back to the Stockholm conference and recurs as an element of the politics and sovereignty trajectory of GEG.

*Pathway 3: Integration*. Since the first ideas about global governance on the planetary scale in the 1970s, there has been a desire among scientists, environmentalists, and decision makers to include more elements in the environment. As in the case with the environment-equity link, this expansion was part of the dispersion. The response has repeatedly been to articulate more comprehensive and integrative approaches. One example is a consequence of the growing understanding of the significance of the Earth System. What once was known as ‘environmental policy’ has thus expanded and is now sometimes called ‘Earth System Governance’ (Biermann 2012, 2014; Biermann and Kim 2020). The emergence and growth of the Earth System approach links quite closely to the rise of the notion of the Anthropocene and the Planetary Boundaries framework, and has received a sizable following. This approach also has the same limitations. Its strength lies in the scientific parameters that characterize the boundary conditions, but it has so far offered less when it comes to the actual policy conclusions. Merging the social and the environmental/scientific remains a challenge.

*Pathway 4: Eco-modernization*. This has roots in the 1980s and especially the Brundtland Commission (1987), the idea that states and other actors should combine traditional policy goals such as economic growth, social welfare, defense and security, with environmental and other SDG imperatives. Eco-modernization still appears to influence policy. The ‘good Anthropocene’ is also now part of its repertoire demonstrating a certain adaptive capacity of the concept (Asafu-Adjaye et al. 2015; a critique in Hamilton 2016). A specific dimension of it is to mobilize the private sector. For the plus 50 years that GEG has existed, private corporations have acted both in favor or against environmental initiatives (Elmore 2022; Mody 2023). Action against has been particularly visible in relation to climate denial fueled by fossil fuel companies. An infamous case was the Global Climate Coalition (1989–2001), an advocacy group that opposed limiting greenhouse gas emissions (Brulle 2023). The private sector has in many cases pushed for voluntary schemes instead of strict environmental regulations and it has been a strong voice for thinking in terms of win–win solutions. With the growing likelihood of major sustainability transformations, firms in multiple sectors have become more and more proactive. An example is the RE100 businesses initiative where hundreds of the world’s largest businesses are pledging to be supplied by 100% renewable electricity by a certain self-chosen date before 2050. Supply-chain pressure exercised by major RE100 members such as Apple may push countries to increase the availability of renewable energy (Liu and Chao 2023). Similar movements have been seen among cities and sub-national regions.

This speaks to the multiple governance approaches taking place. Corporate Social Responsibility (CSR) has been advocated since the 1990s and more recently this engagement has come under the name of ‘Environmental, Social, and Governance’ (ESG) with a focus on investment and reporting aligned with Agenda 2030 goals. Whether greenwashing or not, the language of CSR and ESG have penetrated the corporate sphere and demonstrates the dispersal of GEG and the normalization of the environment. It illustrates global coordination of environmental issues below the international level. *Eco-modernization* and CSR/ESG relate broadly to neoliberalization and to notions of green capitalism, and illustrate the significant, and often under-estimated power of the corporate sector over GEG progress.

These pathways obviously do not exhaust even all major recent trends in GEG, although they certainly cover some ground. What they do demonstrate is that dispersion is ongoing, while the institutional core that early seminal proponents of a supra global structure wanted to see, still only exists in a vague form (e.g., Åström 1972; Strong 1973).

## Concluding reflection

### Implications of a dispersal view and the rise of a social nature

At the outset of this article, we proposed our ambition to lay out a novel understanding of Global Environmental Governance and we professed to do it through looking at its history. We have tried to demonstrate that a historical approach could help us identify dimensions of GEG that may otherwise easily be overlooked. We have identified a trajectory of dispersal over time, from the micro- and local scales to the planetary, and a proliferation of relevant domains of governance, which we have talked about as governable environmental objects, each with their own history. We have argued that these objects have become ever more numerous and irregular in their behavior, bringing undesired change, risk, threat and ultimately disaster. We have talked about these processes as the performative nature of GEG. The environment as a governance issue is transformed through its own governance, an understanding quite far away from earlier dualistic models of environmental engineering to repair or protect. This means that the search light has been turning increasingly back on ourselves as citizens and societies. Governance has been creative, often in close collaboration with new strands of knowledge that have helped co-create new governable objects.

We have also argued that this has led to an omnipresence, or normalization of the environmental in our social and political fabric. In that sense, it could be stated that the environment as a governance undertaking has been phenomenally successful. The relationship between humanity and the planet is both profound and inevitable. The nature Western civilization once wanted to “tame” we are instead accelerating at an ever-growing pace. The histories we tell of ourselves are increasingly becoming elemental, tracking the growing amplitudes of gases and liquids, air and water, ice and snow, soils and plants. The effects of the acceleration are often dire. Yet, we can’t see how this will change soon in any major way, although GEG is aimed at least partly to do just that, assist in making societies do the right thing. So, global governance leaves us with an ever growing and dispersing amount of policy objects, and proxies with which to monitor them and boundaries and goals to avoid or to reach for them. That is the great dispersal that we have tried to describe. But, as humanity and as aggregates of actors, we seem less capable to provide the kind of hands on governance that is required to decisively reduce risk and impacts in the near term. To see both these faces of GEG is perhaps a helpful outcome of our approach, in its own right. Under any circumstances, we seem to be going ever deeper into a period in history where the human-earth relationship will only grow in importance, although we do not know what direction will be taken.

We may think about the prospects of GEG as changed by the rise of the concept of the Anthropocene since the beginning of this century. When humanity, as a collective, finds itself as the largest single geological force on earth and constantly extends its hegemony, the very word ‘governance’ stands against phenomena on a scale hitherto unknown to human responsibility. The kinds of achievements and desires societies have strived toward, for generations and centuries of territorial expansion and resource extraction, may actually become ambitions societies will seek to temper or avoid. Governance may be about *not* doing things we used to do, and certainly about transformation. It may also be about navigating in a more cynical, denialist direction. We don’t know what forces will prevail.

Most of the existing discourse about GEG has had a benevolent trajectory as its premise; not just a path toward sustainability, but a progressive path. Governance was always assumed to improve. Nations of the world have agreed on the 17 SDGs, and pledged to reach their goals. But what if we fail, as it seems we will, and what if actors around the world, big and small, don’t care much? What would global environmental governance be then? Would it have lost its energy? A historical approach may serve as a resilient version of the concept, and the narrative. Making sense of change is meaningful regardless of the directions that the multifarious and dispersed environmental agencies, in a huge plural, take in the future.

If read against the backdrop of the longer history of global governance efforts, it is hard not to see the four pathways we sketched above mostly as variations on themes that were developed some time ago, some even in the very early years of the rise of the environment as a global issue. This may seem a depressing conclusion, and we cannot rule out the possibility that institutional breakthroughs that have not happened so far may still come in the future. It will nonetheless be useful to regard the slow and evolutionary pace of change, despite hopes of the forthcoming green transformation on the global scale. A reflection of the daunting size of the policy challenges the world still faces. Governing the human-planetary relation with a global population approaching ten billion people and with a size of the world’s economy more than a hundred times bigger than it was in 1900 is, obviously, unprecedented.

The situation is paradoxical. On the one hand, the environment has grown in every possible direction and become an extended natural version of society. It could perhaps be described as an extensive zone where the human enterprise, we can call it ‘societies’, encounters nature and draws the natural into the social sphere. That is what the great dispersal has wrought: *a social nature of human responsibility*, and that is, we might say, in and of itself a progress of historical proportions. We engage in issues now that just a couple of generations ago did not exist to us, despite the fact that they existed out there. This is an ongoing evolution and the above set of mega-trends, or others that one might propose, do not alter this overarching pattern. On the contrary, they reinforce and prolong it.

This way of looking at environmental policy echoes some of the basic premises frequently discussed by Bruno Latour (1993, 2004). Environment, climate and biopolitics cannot be disentangled and labeled as either ‘natural’ or ‘social’; they are both. To say that the environment has become more social, or even a part or an extension of society, is according to this line of thought more than just a turn of phrase. Looking at global environmental governance in an empirical and historical way has turned our thinking along a similar trajectory. However, GEG in its immensity does not have any conventional or standardized expertise in the way that particular domains within it, such as pollution, species loss, and melting tundra have expertise. Instead, GEG has augmented into a complex, sprawling enterprise with both civic and democratic properties.

This was a projected or even a desired development from the first visions of a global governance structure in the 1960s and 70s. Other social concerns—legitimate enough: security, wealth, education, public health—tend however to outweigh the equally social concerns that are environmental. We may as we did throughout this article, call this a *normalization* of global environmental governance, and perhaps of the state of the environment itself. Originating in nature—something modernity placed distinctly outside of the human—the environment is now essentially something that we cannot cease to include in our thinking about ourselves, as human, in the world. We could say that the discovery side of governance has been working well. What has not been working so well is the response and intervention side.
